# Development and internal validation of a machine learning model for predicting intracranial infection after spontaneous intracerebral hemorrhage: a two-center retrospective study

**DOI:** 10.3389/fneur.2026.1835984

**Published:** 2026-05-04

**Authors:** Yizhao Lin, Wentong Zheng, Dankui Zhang, Chaoying Wang

**Affiliations:** 1 Department of Laboratory Medicine, Dehua County Hospital, Quanzhou, China; 2 Department of Neurosurgery, Second Affiliated Hospital of Fujian Medical University, Quanzhou, China

**Keywords:** intracerebral hemorrhage, intracranial infection, light gradient boosting machine, machine learning, predictive model, prognosis

## Abstract

**Background:**

Intracranial infection (ICI) is a serious complication following spontaneous intracerebral hemorrhage (ICH) and is associated with prolonged intensive care, increased morbidity, and poor functional outcomes. Early identification of patients at high risk for post-ICH ICI remains difficult because of heterogeneous clinical presentations and complex interactions among neurological severity, systemic inflammation, and treatment-related factors. This study aimed to develop and validate a clinically applicable machine learning model for early prediction of ICI after ICH.

**Methods:**

This two-center retrospective study included 1,317 patients with spontaneous ICH admitted to two hospitals in the same province, between 2015 and 2024. Baseline demographic, clinical, laboratory, and radiological variables obtained within 24 h of admission were used to construct the prediction models. Twelve machine learning algorithms were compared, and a Light Gradient Boosting Machine (LGBM) model demonstrated the best overall performance. Model discrimination, calibration, and clinical utility were evaluated using receiver operating characteristic analysis, calibration plots, precision–recall curves, decision curve analysis, and 10-fold cross-validation. Associations between model-predicted risk, ICI occurrence, and 180-day functional outcomes were assessed.

**Results:**

Intracranial infection occurred in 165 patients (12.5%). The LGBM model showed excellent test-set discrimination (AUC = 0.923), and supplementary 10-fold cross-validation on the overall cohort suggested relatively stable performance across folds (mean AUC = 0.933). Higher model-predicted risk was independently and nonlinearly associated with increased ICI risk and was significantly associated with unfavorable 180-day functional outcomes.

**Conclusion:**

This ML model showed good performance for the early prediction of ICI after ICH using routinely available clinical data and may support risk stratification in neurocritical care settings. However, because only internal validation was performed, further external validation is needed before broader clinical application.

## Background

Spontaneous intracerebral hemorrhage (ICH) is one of the most lethal and disabling stroke types (1). This is due to its sudden onset (parenchymal hemorrhage), early case fatality, high risk of disability, and long-term neurological impairment, which ultimately impose major societal and family burdens (2, 3). Population-based and hospital-based studies indicate that a high proportion of ICH survivors either do not survive, or become dependent after the 1 year mark, with functional outcomes being tightly associated to baseline hematoma especially when intraventricular hemorrhage is also present, and perihematomal edema volumes (4, 5). In China, a high-incidence country, recent national registry data collected from more than 7,000 surgically managed patients confirmed persistently high short-term mortality and poor functional recovery rates, revealing ICH as a heavy healthcare and economic burden (6). In addition to mortality, ICH patients who survive experience long-term neuropsychiatric and cognitive complications, such as depression, anxiety, and cognitive deficits, which further impair their quality of life and caregiver burden (7). These unfavorable outcomes are not only attributable to primary hematoma, but are also driven by secondary injury processes, such as systemic and central nervous system (CNS) inflammation (8). Among in-hospital complications after ICH, intracranial infection (ICI) is clinically important because it is associated with prolonged intensive care and unfavorable neurological outcomes (9). These data demonstrate that ICH has a disproportional burden of mortality, disability, and long-term neuropsychiatric morbidity and suggest that early recognition of ICH patients at risk of post-ICH ICI is key to improving prognosis and driving targeted prevention strategies (2–4).

ICI is a common complication of ICH; not only does it have devastating consequences but its early prediction remains difficult. Current clinical practice mainly relies on CSF culture and nonspecific inflammatory markers, but these approaches are constrained by delayed turnaround time, limited sensitivity, and insufficient ability to support early individualized intervention (10). In addition, clinical prediction models for patients with external ventricular drainage (EVD) have been constructed based on logistic regression; however, their variable selection and feature weighting are still performed manually, limiting their generalizability and clinical utility. For instance, Fu et al. identified operation time, albumin level, and a history of diabetes as risk factors for ICI use among patients on EVD (11). Furthermore, most previous studies were performed at single institutions with relatively small sample sizes and lacked multicenter external validation to demonstrate their translational capability (2, 12). Finally, traditional statistical methods are inadequate for modelling nonlinear interactions among perioperative, biochemical, and inflammatory factors that may jointly determine the risk of postoperative infection (13). Machine learning (ML) methods can overcome these limitations by utilizing high-dimensional data to discover latent associations that can be incorporated into personalized risk prediction models (14). Therefore, this study was designed to develop an ML prediction model to identify patients with ICH at high risk of ICI as early as possible, providing an actionable decision support tool for clinicians to intervene and manage patients more precisely.

This study aimed to develop and internally validate an ML model for predicting ICI after ICH using a two-center clinical dataset. The Light Gradient Boosting Machine (LGBM) algorithm was used to establish the model by integrating baseline demographic, clinical, laboratory, and radiological variables available within 24 h of admission to fit complex nonlinear relationships. The association between the model-predicted risk and 180-day functional outcomes was also evaluated to facilitate clinical interpretation. It can be used as a novel data-driven tool for the early risk stratification and outcome-oriented management of neurocritical care patients.

## Methods and materials

### Studying population selection and study workflow

**Figure 1:**
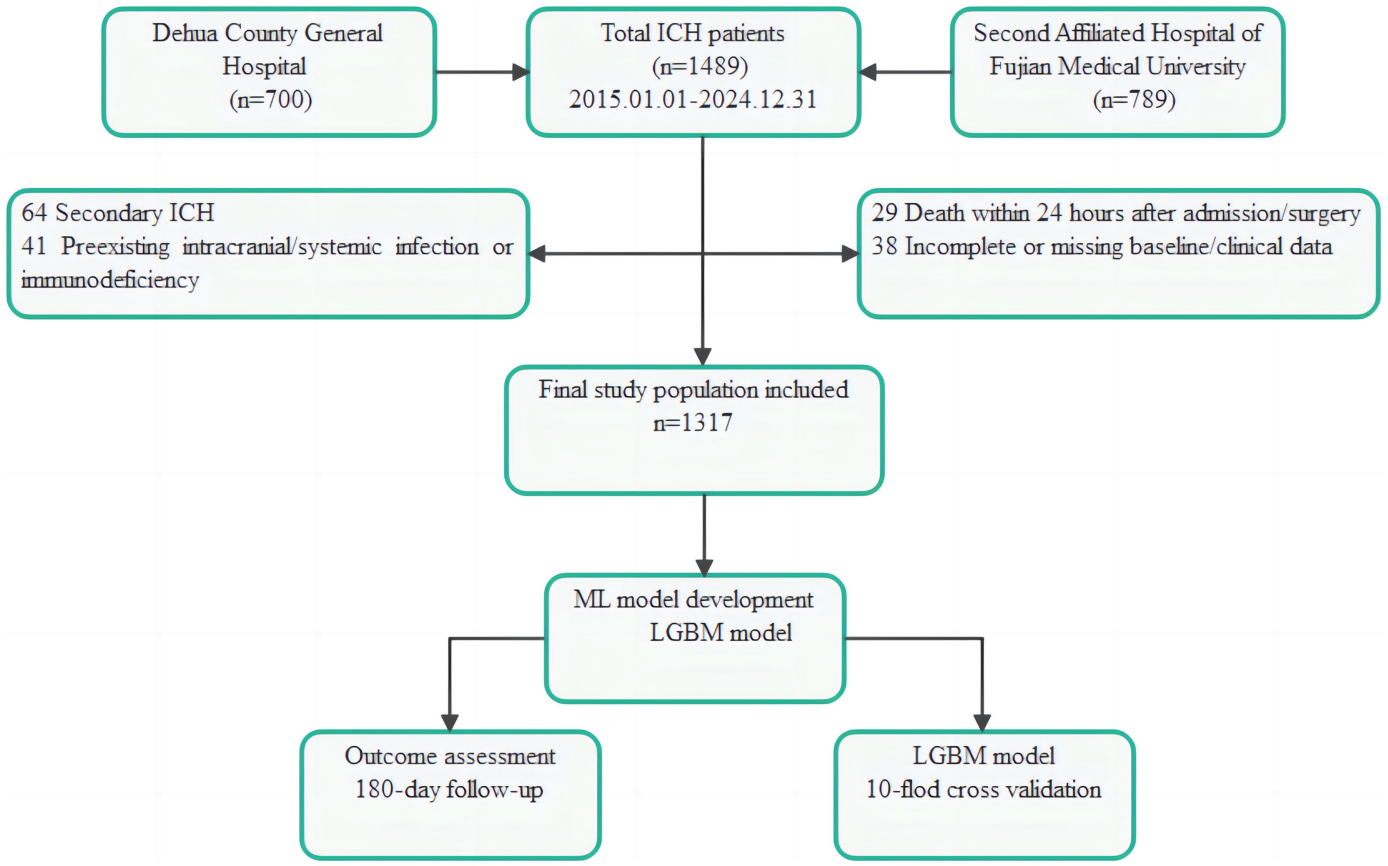
Flowchart of patient selection and study design. Overall, 1,489 patients with spontaneous ICH admitted to Dehua County General Hospital (*n* = 700) and Second Affiliated Hospital of Fujian Medical University (*n* = 789) from January 1, 2015, to December 31, 2024, were screened. After excluding 64 patients with secondary ICH, 41 patients with preexisting intracranial/systemic infection or immunodeficiency, 29 patients who died early (within 24 h after admission or surgery), and 38 patients with incomplete or missing baseline/clinical data, 1,317 eligible patients were included in the final analysis. An LGBM model was then developed to predict post-ICH intracranial infection, followed by 10-fold cross-validation and 180-day functional outcome assessment using mRS.

Patients with spontaneous ICH enrolled in this study were admitted to the Dehua County Hospital and the Second Affiliated Hospital of Fujian Medical University between January 1, 2015, and December 31, 2024. A total of 1,489 patients were screened for this study (Figure 1). Recruitment occurred according to the following inclusion criteria: (1) Diagnosis of spontaneous ICH was confirmed by cranial computed tomography (CT); (2) Age ≥ 18 years; (3) Admission to hospital within the study period with baseline clinical, laboratory and imaging data within 24 h after admission; (4) Receiving surgical or conservative treatment; (5) Completed follow-up for 180 days after ICH onset, and the Modified Rankin Scale (mRS) was used to evaluate the functional outcome. Patients were excluded according to the following exclusion criteria: (1) Secondary ICH caused by trauma, tumor, vascular malformation, aneurysm rupture, hemorrhagic transformation of ischemic stroke, or other reasons; (2) Preexisting intracranial or systemic infection or known immunodeficiency before admission; (3) Death within 24 h after admission or death due to surgical failure; (4) Incomplete baseline data or insufficient data to support model analysis; (5) Recurrent ICH during the same hospitalization or other severe neurological diseases (such as subarachnoid hemorrhage, encephalitis, and brain abscess) that could affect prognosis during the follow-up period.

After excluding patients based on predefined exclusion criteria, 172 patients were excluded: secondary ICH due to trauma, tumor, vascular malformation, aneurysm rupture, or hemorrhagic infarction (*n* = 64); preexisting ICI, systemic infection, or immunodeficiency (*n* = 41); early death within 24 h after admission or surgery (*n* = 29); and incomplete baseline or clinical data (*n* = 38). Finally, 1,317 patients were included in the final cohort for analysis. The overall dataset was divided into a training set and a test set for model development and performance assessment. An LGBM model was developed to predict post-ICH ICI, with model fitting performed on the training set and performance evaluated in the test set. In addition, 10-fold cross-validation was conducted on the overall cohort as a supplementary internal robustness analysis. The clinical outcomes at 180 days were evaluated to determine functional prognosis using the mRS.

### Data collection, diagnostic and outcome assessment

Baseline demographic, clinical, laboratory, and radiological data obtained within 24 h of admission, including age, sex, comorbidities (hypertension, diabetes, smoking, and drinking), GCS, hematoma volume, electrolytes, inflammatory markers, and hematologic indices, were obtained from the electronic medical record system and considered as candidate predictors for model development.

The diagnosis of ICI after ICH was defined according to the CDC criteria and the Chinese Guidelines for the Diagnosis and Management of Spontaneous ICH (15–17), based on a compatible clinical presentation (such as fever, altered consciousness, or meningeal irritation), laboratory or CSF abnormalities (such as increased leukocytes, elevated protein, decreased glucose, or positive culture), and/or neuroimaging findings suggestive of infection (such as ventricular debris or ependymal enhancement). In the present study, post-ICH ICI was defined as intracranial infection diagnosed more than 48 h after hospital admission to ensure adequate temporal separation from baseline predictor collection.

The 180-day functional outcome was measured with mRS, which was scored by trained clinicians who were blinded to infection status and model predictions through a standardized outpatient interview or telephone follow-up. An mRS score of 0–2 was considered a favorable outcome (functional independence), and an mRS score of 3–6 was considered an unfavorable outcome (death or disability).

### Treatment procedure

Treatment (Surgical or conservative) was performed according to the Chinese guidelines for the diagnosis and management of spontaneous Intracerebral Hemorrhage (16, 17). Surgical indications were a hematoma volume > 30 mL or a significant midline shift, intraventricular extension with hydrocephalus, and rapid neurological deterioration. Surgical treatment methods mainly include craniotomy hematoma evacuation, minimally invasive aspiration, or external ventricular drainage, according to the location of the hematoma and the patient’s condition. Patients without surgical indications received standardized conservative treatment, including blood pressure control, hemostasis, dehydration therapy, and prevention of secondary complications.

### Ethics statement

This study was approved by the Ethics Committees of Dehua County General Hospital [Approval No. 2023 L (011)] and the Second Affiliated Hospital of Fujian Medical University [Approval No. (2022) 35] and conducted in accordance with the Declaration of Helsinki (2013 revision). This study was exempt from the need to obtain informed consent because of its retrospective design and anonymized data collection. All the patient data were kept confidential.

### Statistical analysis

**Figure 2:**
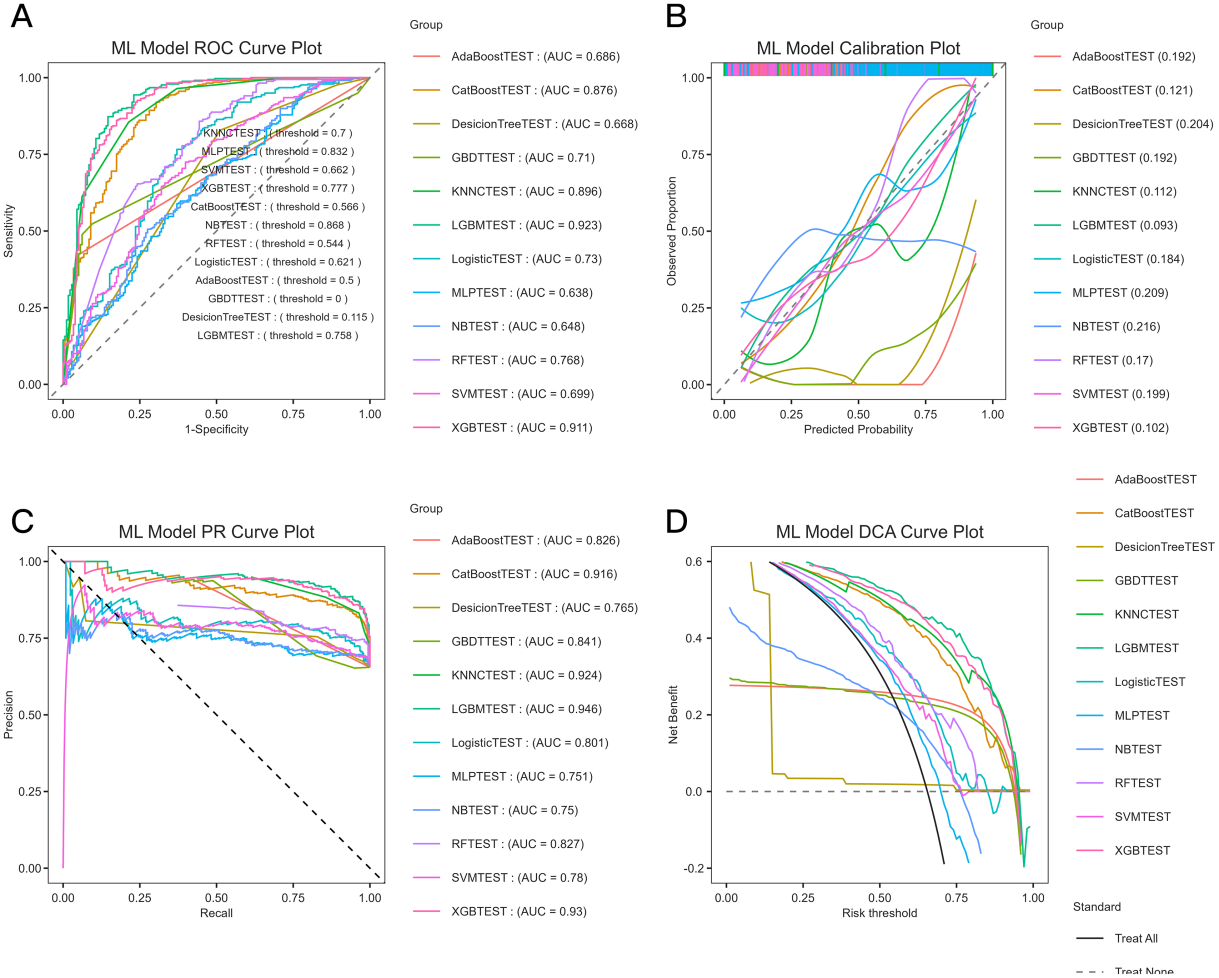
ML performance in the 12 models with the best feature selection results for predicting ICI after ICH. **(A)** Receiver operating characteristic (ROC) curves of each model. The AUC of LGBM model was the largest (AUC = 0.923), and XGBoost (AUC = 0.911) and CatBoost (AUC = 0.876) ranked the second and third, respectively. **(B)** Calibration curves of predicted and observed probabilities for the 12 models. LGBM and XGBoost models had the best calibration, with their predicted lines being closest to the ideal 45° line. **(C)** Precision–recall (PR) curves for each model. The AUC of LGBM model was the largest (AUC = 0.946), followed by XGBoost (AUC = 0.911) and CatBoost (AUC = 0.886). **(D)** Decision curve analysis (DCA) for each model. LGBM and XGBoost models provided the highest net benefit for a wide range of threshold probabilities, indicating their superior clinical applicability.

Continuous variables were summarized as mean ± standard deviation or median (interquartile range) and compared using Student’s t-test or Mann–Whitney U test, as appropriate. Categorical variables were presented as counts and percentages and were compared using the χ^2^ test or Fisher’s exact test, as appropriate. Feature selection was performed using the Boruta algorithm, and multicollinearity was evaluated using the variance inflation factor (VIF). The overall dataset was divided into a training set and a test set. Post-ICH ICI were predicted using the LGBM model. The model was constructed using the selected features with the default hyperparameter settings implemented in the corresponding package, unless otherwise specified. Because the incidence of ICI was relatively low, a SMOTE-based oversampling strategy was applied during model development to reduce the impact of class imbalance. Model fitting was performed in the training set, and model performance was assessed in the test set. In addition, 10-fold cross-validation was conducted on the overall cohort as a supplementary internal robustness analysis. Because this cross-validation procedure was not implemented as a fully nested feature-selection framework in which Boruta was repeated independently within each fold, the corresponding cross-validation results were interpreted cautiously. Model discrimination was assessed by the area under the receiver operating characteristic (ROC) curve (AUC). Calibration plots, precision–recall (PR) curves, and decision curve analyses (DCA) were also performed to evaluate calibration and clinical utility. Nonlinear relationships between the LGBM-predicted probabilities and the risk of post-ICH ICI were explored using restricted cubic spline (RCS) regression. Logistic regression models were used to analyze trends, and adjustments were made for potential confounders. The ROC, calibration, PR, and DCA were used to comprehensively evaluate the predictive ability of the 12 models (Figure 2).

All analyses were performed using the R software (version 4.3.2) and Python (version 3.11). Statistical significance was set at *p* < 0.05.

## Result

### Baseline demographic and clinical characteristics of the study population

In the final analysis, 1,317 patients with spontaneous ICH were enrolled, and 165 (12.5%) developed ICIs during hospitalization. As shown in Table 1, patients with infection were younger than those without infection (56.34 ± 11.53 vs. 58.57 ± 11.44, *p* = 0.021). There were no significant differences in sex, history of hypertension, diabetes, smoking, or alcohol use between the two groups (*p* > 0.05).

**Table 1:** The study population baseline characteristics.

Variable	Overall	Non-ICI	ICI	*p*-value
	*N* = 1317	*N* = 1152	*N* = 165	
Age	58.29 ± 11.47	58.57 ± 11.44	56.34 ± 11.53	0.021
Gender, *n* (*p*%)				0.573
Female	481.00 (36.52%)	424.00 (36.81%)	57.00 (34.55%)	
Male	836.00 (63.48%)	728.00 (63.19%)	108.00 (65.45%)	
HP, *n* (*p*%)				0.607
No	322.00 (24.45%)	279.00 (24.22%)	43.00 (26.06%)	
Yes	995.00 (75.55%)	873.00 (75.78%)	122.00 (73.94%)	
DM, *n* (*p*%)				0.962
No	1,135.00 (86.18%)	993.00 (86.20%)	142.00 (86.06%)	
Yes	182.00 (13.82%)	159.00 (13.80%)	23.00 (13.94%)	
Somking, *n* (*p*%)				0.375
No	1,043.00 (79.20%)	908.00 (78.82%)	135.00 (81.82%)	
Yes	274.00 (20.80%)	244.00 (21.18%)	30.00 (18.18%)	
Drinking, *n* (*p*%)				0.348
No	1,155.00 (87.70%)	1,014.00 (88.02%)	141.00 (85.45%)	
Yes	162.00 (12.30%)	138.00 (11.98%)	24.00 (14.55%)	
Time to onset, mean ± sd	6.33 ± 10.27	6.61 ± 10.89	4.42 ± 3.09	<0.001
GCS, mean ± sd	10.18 ± 3.47	10.36 ± 3.48	8.92 ± 3.15	<0.001
K, mean ± sd	3.84 ± 10.74	3.89 ± 11.48	3.42 ± 0.50	0.166
Na, mean ± sd	137.99 ± 6.06	137.95 ± 6.31	138.24 ± 3.90	0.417
Ca, mean ± sd	2.32 ± 0.17	2.32 ± 0.17	2.35 ± 0.17	0.011
P, mean ± sd	1.00 ± 2.42	1.02 ± 2.59	0.87 ± 0.34	0.072
Mg, mean ± sd	0.86 ± 0.21	0.86 ± 0.23	0.84 ± 0.10	0.030
GLU, mean ± sd	9.00 ± 25.75	9.06 ± 27.51	8.57 ± 2.83	0.558
Albumin, mean ± sd	39.90 ± 5.26	39.89 ± 5.31	39.96 ± 4.87	0.866
WBC, mean ± sd	10.59 ± 4.45	10.48 ± 4.42	11.34 ± 4.60	0.025
Neutraphil, mean ± sd	8.63 ± 4.35	8.49 ± 4.30	9.58 ± 4.55	0.004
Lymphocyte, mean ± sd	1.37 ± 0.98	1.39 ± 1.01	1.23 ± 0.80	0.021
Monocyte, mean ± sd	0.52 ± 0.38	0.52 ± 0.40	0.51 ± 0.27	0.452
Hb, mean ± sd	147.02 ± 22.37	146.34 ± 21.89	151.76 ± 25.07	0.009
PLT, mean ± sd	200.13 ± 79.41	201.80 ± 81.38	188.47 ± 62.97	0.015
Hemorrhage volume, mean ± sd	28.55 ± 23.62	27.77 ± 23.36	34.01 ± 24.77	0.003
LOS, mean ± sd	19.95 ± 12.96	19.06 ± 12.00	26.14 ± 17.10	<0.001
Treatment, *n* (*p*%)				<0.001
Non-surgery	659.00 (50.04%)	602.00 (52.26%)	57.00 (34.55%)	
Surgey	658.00 (49.96%)	550.00 (47.74%)	108.00 (65.45%)	
MRS group, *n* (*p*%)				<0.001
Favourable	623.00 (47.30%)	572.00 (49.65%)	51.00 (30.91%)	
Unfavourable	694.00 (52.70%)	580.00 (50.35%)	114.00 (69.09%)	

ICI, intracranial infection; WBC, white blood cell; K, kalium; Na, sodium; Ca, calcium; P, phosphate; Mg, magnesium; GLU, glucose; Hb, hemoglobin; PLT, platelets; DM, diabetes mellitus; HP, hypertension; LOS, length of stay; MRS, Modified Rankin Scale; GCS, Glasgow Coma Scale.

Differences in the neurological severity and acute disease conditions were also observed. Compared with non-infected patients, patients with infection had a lower GCS score on admission (8.92 ± 3.15 vs. 10.36 ± 3.48, *p* < 0.001) and a shorter onset-admission time (4.42 ± 3.09 vs. 6.61 ± 10.89, *p* < 0.001). In terms of electrolyte and biochemical abnormalities, patients with infection had higher serum calcium levels (2.35 ± 0.17 vs. 2.32 ± 0.17, *p* = 0.011) and lower magnesium levels (0.84 ± 0.10 vs. 0.86 ± 0.23, *p* = 0.030) than those without infection. Inflammatory indicators were higher, with white blood cell counts (11.34 ± 4.60 vs. 10.48 ± 4.42, × 109/L, *p* = 0.025) and neutrophil counts (9.58 ± 4.55 vs. 8.49 ± 4.30, × 109/L, *p* = 0.004) being higher and lymphocyte counts being lower (1.23 ± 0.80 vs. 1.39 ± 1.01, × 109/L, *p* = 0.021). Regarding hematological variables, patients with infection had higher hemoglobin (*p* = 0.009) and lower platelet levels (*p* = 0.015). Length of Stay (LOS), Treatment, and MRS group are presented in Table 1 to provide a complete descriptive profile of the study population and outcomes, but these variables were not included as candidate predictors in the model-building process.

### Feature selection and model construction

**Figure 3:**
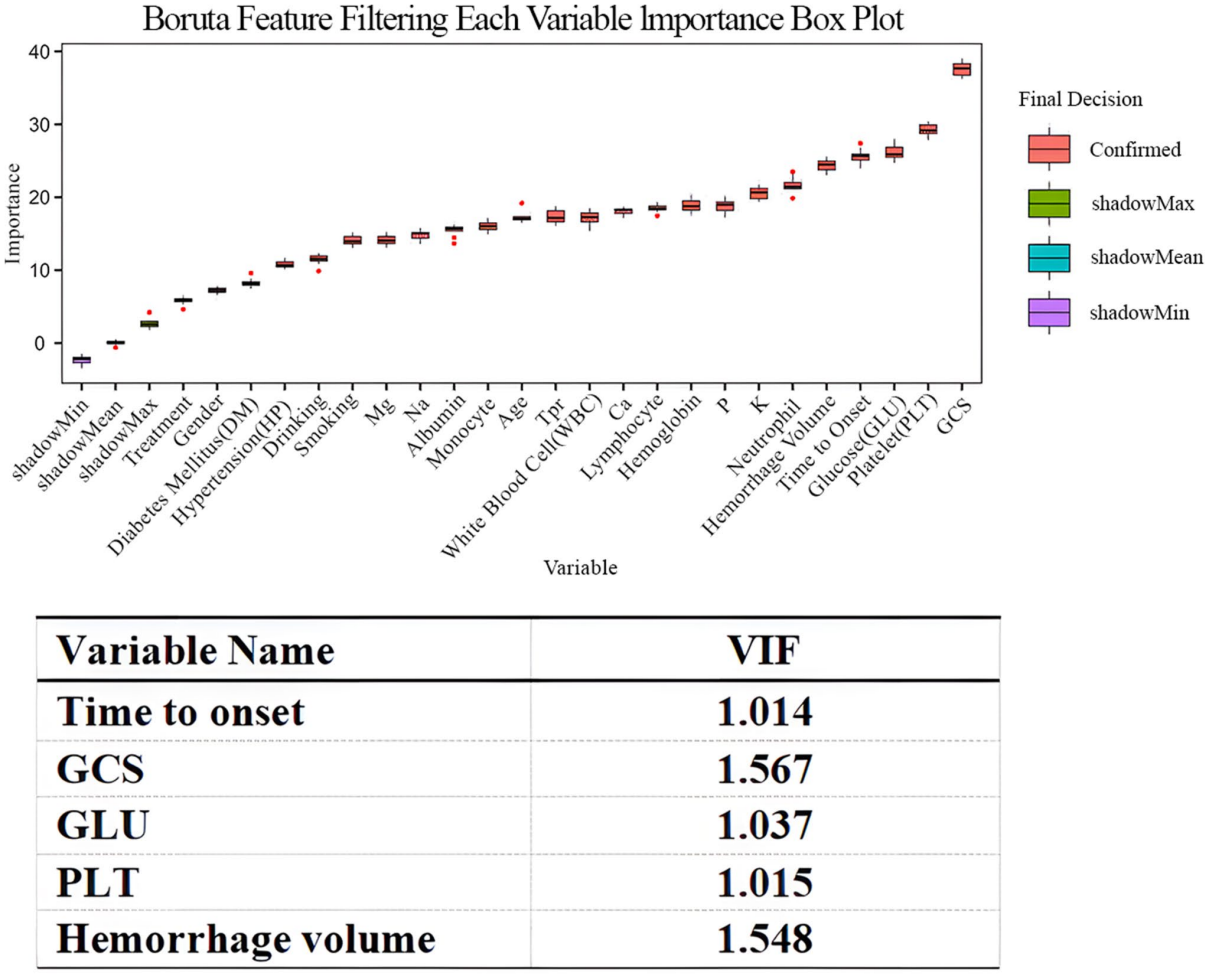
Feature selection and multicollinearity analysis of variables in the machine learning model. The Boruta algorithm was used to select the most important predictors of intracranial infection following ICH. The box plot represents the distribution of importance scores for each variable, with red boxes indicating the features selected as significant by the Boruta algorithm. GCS, hemorrhage volume, PLT, GLU, and time to onset had the highest importance. The variance inflation factor (VIF) was used to detect multicollinearity, and all variables were <2, suggesting no significant collinearity between the features. These variables were used in the LGBM model to predict post-ICH intracranial infection.

Boruta feature selection was performed to identify the most informative variables for predicting ICI after ICH. As shown in Figure 3, GCS, hemorrhage volume, PLT, GLU, and time to onset were identified as the most important predictors and were retained for subsequent model construction. Multicollinearity assessment showed that all selected variables had VIF values < 2, indicating no significant collinearity among the retained features. These variables were then incorporated into the LGBM model for prediction of post-ICH intracranial infection.

### Model performance and clinical usefulness

**Figure 4:**
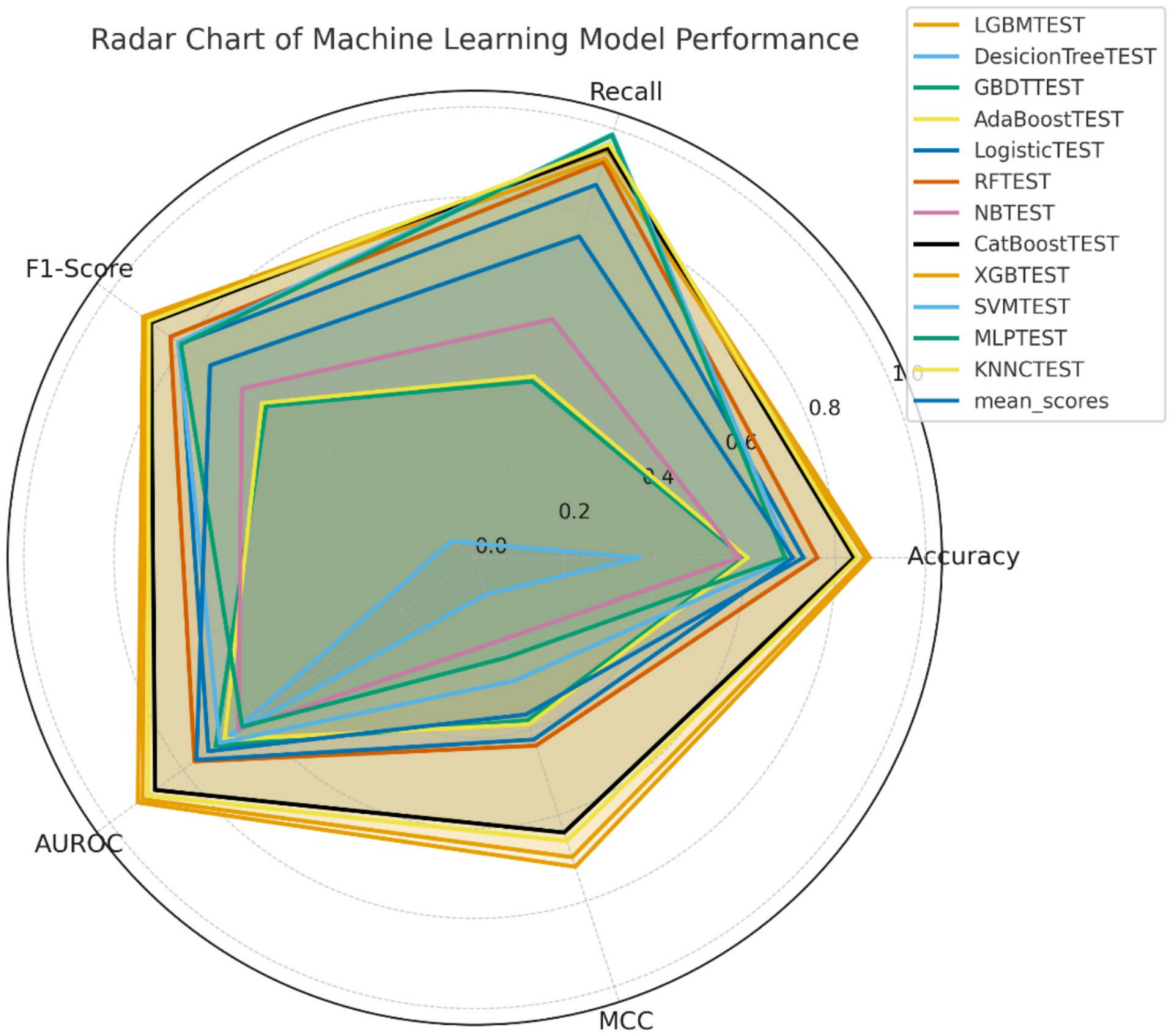
Radar chart of the ML model’s prediction results for intracranial infection post-ICH. The radar chart reflects the performance of 12 machine learning algorithms with 5 indicators (accuracy, recall, F1-score, AUROC, MCC). It can be seen that among the 12 machine learning algorithms, the area covered by LGBM was the largest and most uniform, which means that its overall performance is the best and the performance in all aspects is relatively stable. At the same time, the area of the conventional decision tree and Naïve Bayes is the smallest, which also means that the performance in some evaluation metrics is not good. In general, the area of coverage of the ensemble learning algorithms, such as LGBM, XGBoost, and CatBoost, is larger than that of the other algorithms, which also shows that the former has better performance, stability, and generalization ability.

ROC, calibration, PR, and DCA were used to comprehensively evaluate the predictive ability of the 12 models (Figure 2). In the test-set evaluation, LGBM achieved the highest AUC (0.923), followed by XGBoost (AUC = 0.911) and CatBoost (AUC = 0.876), indicating superior discriminatory performance among the compared models. In addition to AUROC, the LGBM model showed a sensitivity of 95.0%, specificity of 73.6%, positive predictive value (PPV) of 87.2%, and negative predictive value (NPV) of 88.6% under the oversampling-based evaluation framework used in this study (Figure 4). The same trend was observed in the calibration analysis, with both the LGBM and XGBoost models exhibiting the best calibration. Moreover, in PR analysis, LGBM had the largest AUC value (0.946). Finally, the LGBM and XGBoost models exhibited the widest DCA ranges and provided the most significant net benefits, suggesting their superiority in clinical applicability for individualized prediction and decision-making based on risk. A SHAP-based interpretability analysis performed in the testing set further showed that GCS, PLT, GLU, hemorrhage volume, and time to onset were the main contributors to the LGBM model predictions (Supplementary Figure S1). The predictors used in this model were all drawn from data routinely available in electronic medical records, suggesting that the model could be integrated into existing hospital information systems without the need for extra data collection.

### Supplementary cross-validation and robustness analysis

**Figure 5:**
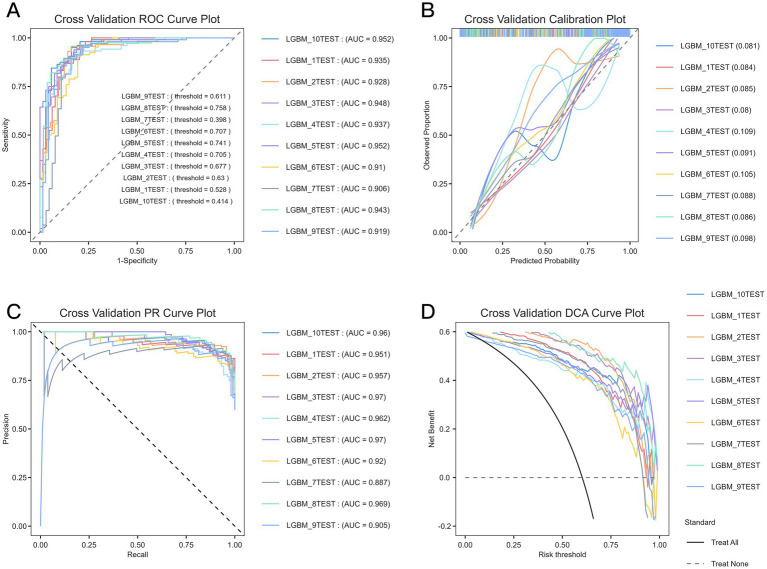
Internal validation of LGBM model performance by 10-fold cross-validation. **(A)** ROC curves of 10-fold cross-validation showed high consistency of the discriminative ability among all 10 folds (mean AUC = 0.933, range: 0.906–0.952). **(B)** Calibration curves of 10-fold cross-validation revealed that the predicted probabilities of intracranial infection were highly consistent with the observed outcomes in each fold. **(C)** PR curves of 10-fold cross-validation showed high precision and recall in all folds (mean PR-AUC = 0.946). **(D)** DCA of 10-fold cross-validation showed that the LGBM model provided net benefit and wide applicability at a range of threshold probabilities in each fold.

The supplementary 10-fold cross-validation results of the LGBM model are shown in Figure 5. Across the 10 folds, the model showed relatively stable performance (mean AUC ± standard deviation, 0.933 ± 0.019; range, 0.906–0.952). The calibration curves suggested acceptable agreement between predicted and observed risk, and the PR curves showed consistently high predictive precision, with a mean PR-AUC of 0.946. DCA indicated a net clinical benefit across a range of threshold probabilities. However, because this cross-validation analysis was conducted on the overall cohort rather than within a fully nested feature-selection framework, these findings should be interpreted as a supplementary internal robustness assessment under the current workflow, rather than as an unbiased estimate of generalizable performance.

### Nonlinear association between the predicted value of the LGBM model and the risk of ICI after ICH

**Figure 6:**
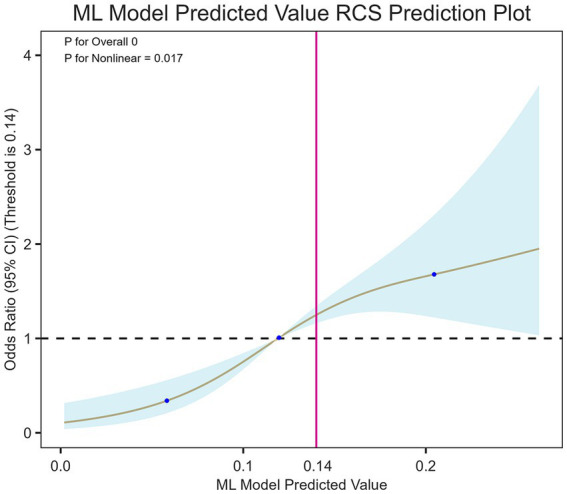
RCS of the association between the predicted value of the LGBM model and the risk of ICI after ICH. The restricted cubic spline (RCS) model for the nonlinear association between the Light Gradient Boosting Machine (LGBM) model-predicted probability and the risk of intracranial infection after intracerebral hemorrhage (ICH). The solid brown line is the estimated odds ratio (OR) and the shaded blue area is the 95% confidence interval (CI). The vertical magenta line is the optimal cutoff of 0.14. A statistically significant nonlinear relationship was found between the predicted value and the intracranial infection after ICH (*P* for nonlinearity = 0.017).

Figure 6 shows that when RCS was used, there was a significant nonlinear correlation between the value predicted by the LGBM model and the risk of ICI after ICH (P for nonlinearity = 0.017). When the predicted probability value was low, the risk of ICI after ICH was low, but gradually increased. When the predicted probability exceeded the threshold value (the threshold value is shown by the dotted line), the Odds Ratio (OR) increased sharply, indicating that the predicted values were higher for patients with ICI. In general, the LGBM has a certain accuracy in learning and simulating nonlinear relationships.

### Exploratory analysis of LGBM-predicted risk quartiles and observed ICI risk

As shown in the revised Table 2, when patients were stratified according to quartiles of the LGBM-predicted probability, a progressive increase in observed ICI risk was noted across higher predicted-risk categories. Compared with the lowest quartile (Q1), patients in Q2, Q3, and Q4 showed higher odds of ICI in both unadjusted and adjusted models. This gradient remained statistically significant after adjustment for age and sex, as well as after further adjustment for hypertension, diabetes mellitus, smoking, and drinking. These findings support the ability of the model-derived probability to stratify patients into clinically distinct risk groups, although this analysis was used as an exploratory risk-stratification assessment rather than as a formal validation of model performance.

**Table 2:** LGBM test predicted value trend test.

Exposure	Non-adjusted		Adjust I		Adjust II	
	OR (95%CI)	*P*-value	OR (95%CI)	*P*-value	OR (95%CI)	*P*-value
Predicted value (IQR)						
Q1	1		1		1	
Q2	2.271 (1.227, 4.201)	0.009	2.273 (1.227, 4.209)	0.009	2.264 (1.223, 4.193)	0.009
Q3	3.701 (2.065, 6.632)	<0.001	3.727 (2.078, 6.686)	<0.001	3.738 (2.084, 6.705)	<0.001
Q4	4.484 (2.530, 7.945)	<0.001	4.441 (2.502, 7.883)	<0.001	4.587 (2.582, 8.148)	<0.001
*P* for predicted group trend		<0.001		<0.001		<0.001

Adjust I model adjust for: gender; age.

Adjust II model adjust for: gender; hypertension (HP); diabetes mellitus (DM); smoking; drinking.

OR, odds ratio; CI, confidence interval; IOR, interquartile range.

### Predictive value and prognostic association

**Figure 7:**
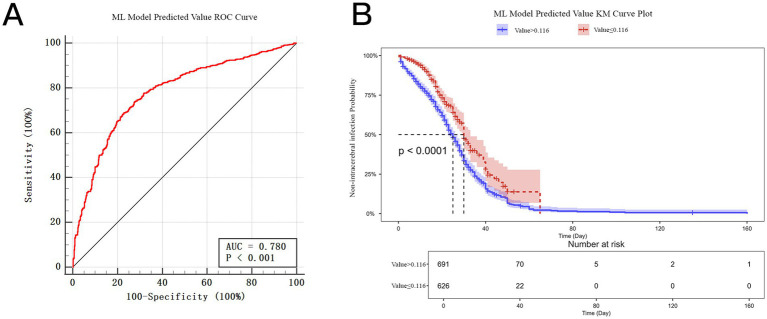
Prediction value and prognostic association of the LGBM model for ICI after ICH. **(A)** The receiver operating characteristic (ROC) curve of LGBM model predicted value for intracranial infection displayed a good discrimination (AUC = 0.780, *p* < 0.001). **(B)** The Kaplan–Meier (KM) curve plotted a significant difference in 180-day survival between patients with ML-predicted risk above or below the optimal threshold value (0.116). The patients with predicted values ≤ 0.116 had significantly better survival than those > 0.116 (*p* < 0.0001, log-rank test). The shaded areas represent 95% confidence intervals. A higher ML-predicted value was associated not only with an increased risk of intracranial infection but also with an adverse long-term outcome after ICH.

As depicted in Figure 7A, the probability value predicted by the LGBM had a high predictive performance for the occurrence of ICI after ICH. The ROC curve analysis of the predicted value showed an AUC of 0.780 (*p* < 0.001), indicating good discrimination of the model in identifying patients with and without infection. Moreover, the Kaplan–Meier survival analysis demonstrated a significant association between the predicted value and long-term outcomes. Patients with high predicted probabilities (> 0.116) had a significantly worse 180-day survival rate than those with low predicted probabilities (≤ 0.116) (*p* < 0.0001, log-rank test) (Figure 7B). These findings suggest that an elevated LGBM-predicted value was associated not only with an increased risk of ICI but also with poor functional and survival outcomes after ICH.

## Discussion

In this two-center retrospective study, we developed and evaluated an LGBM model to predict ICI following ICH. In a cohort of 1,317 patients, the model performed better than the other compared ML algorithms, with a test-set AUC of 0.923, while supplementary 10-fold cross-validation on the overall cohort suggested relatively stable performance across folds (mean AUC = 0.933). Most notably, we showed that the LGBM-predicted value was significantly, nonlinearly, and independently associated with infection risk and was strongly associated with worse 180-day functional outcomes, higher mRS scores, and lower survival rates in patients with higher predicted probabilities of infection. These results underscore the ability of the LGBM model to capture complex nonlinear interactions among clinical, laboratory, and surgical variables, and may provide a clinically interpretable and generalizable tool for the early identification of high-risk patients with ICH. This study represents a novel effort to integrate machine learning–based risk stratification into neurocritical care practice to facilitate the improvement of patient prognosis and the development of personalized preventive interventions.

The results of the present study align with and extend those of previous studies on infection-related complications after ICH. Similar to earlier reports, Murthy et al. and Chen et al. identified postoperative or device-related factors such as EVD duration, surgical manipulation, and systemic inflammation as major contributors to ICI (9, 18). Similarly, our model incorporated these variables, confirming their significant contribution to infection risk. Prior biomarker-based studies liked Zhu X et al., including those evaluating CSF heparin-binding protein (HBP) and CD64, demonstrated moderate diagnostic value but were limited by delayed availability and restricted predictive accuracy (10). In contrast, the ML approach integrates multidimensional clinical and laboratory features, enabling earlier risk prediction before ICI develop. Moreover, a recent study on ML predicting postoperative or EVD-associated infections published by Chen et al. reported AUC values of approximately 0.897 (19), however, the study was limited by single-center samples or a lack of prognostic validation. In the two-center cohort, the model demonstrated favorable predictive performance (AUC = 0.923) and showed stable internal validation, while also linking predicted infection risk to long-term functional outcomes, an association previously suggested by Jiang et al., but not quantitatively demonstrated (7). Collectively, these comparisons highlight the advancement of our study over existing evidence by providing a comprehensive and clinically interpretable model that improves the early detection and prognostic assessment of ICIs after ICH.

The predictive power of our LGBM model was underpinned by the strong pathophysiological plausibility of its five key features. This interpretation was further supported by the SHAP-based analysis in the testing set, which quantitatively confirmed that GCS, PLT, GLU, hemorrhage volume, and time to onset were the major contributors to model prediction. First, a lower GCS score, identified as the most important predictor, often signifies more severe initial brain injury and a higher likelihood of requiring invasive procedures such as EVD or prolonged mechanical ventilation, which are established portals of entry for pathogens (20). Second, a larger hematoma volume is not only a marker of injury severity, but also contributes to a more pronounced and prolonged immunosuppressive state post-ICH characterized by impaired lymphocyte function, which predisposes patients to infection (21). Third, the inclusion of PLT count is mechanistically sound, as thrombocytopenia following ICH can be associated with systemic inflammation and has been linked to an increased risk of sepsis and other infections, possibly because of the role of platelets in innate immunity and endothelial integrity (22). Fourth, elevated blood GLU levels, even in non-diabetic patients, create a favorable environment for bacterial proliferation and impair neutrophil and immune cell function (23). Finally, a shorter time from onset to admission may reflect a more rapid and severe clinical deterioration, prompting earlier and more aggressive interventions that inherently carry infection risks (24). Collectively, these variables reflect the neurological severity, systemic inflammatory and immunosuppressive responses, and intensity of care which characterize the LGBM model, providing a coherent biological narrative that strongly supports its clinical validity for predicting post-ICH ICI.

### Limitations and future directions

This model has several limitations. First, this was a retrospective study and was therefore subject to potential selection bias and unmeasured confounding factors despite strict inclusion and exclusion criteria. Data were extracted from electronic medical records that may have been subject to incomplete or inconsistent data entry. Second, although the 10-fold cross-validation demonstrated stable internal performance, no independent center-based external validation was performed. Because the two participating hospitals were located in the same province and the data were pooled for model development, the broader generalizability of the model remains uncertain. Third, the predictive features were limited to routinely collected clinical and laboratory variables. Other potentially relevant predictors, such as surgical details (e.g., operation time and surgical technique), microbiological factors, and novel inflammatory biomarkers, were not included in this model and could potentially improve the predictive performance if available. Finally, the implementation and clinical impact of this model have not yet been tested in practice. The influence of this model on decision-making processes, including prophylactic measures, antibiotic stewardship, and patient outcomes, should be evaluated prospectively. While not externally validated in this study, the use of routinely collected variables and internal cross validation suggests that the model has the potential to be deployed and externally validated in a variety of clinical settings.

Future directions: A prospective multicenter validation of the model in different regions and healthcare systems will strengthen the generalizability of this study. Incorporating novel biomarkers such as inflammatory cytokines, microbiome profiles, and radiomics features from CT or MRI may further improve the predictive performance. Temporal data integration and dynamic prediction models enable real-time risk assessment in neurocritical care. Translating the model into clinical decision support systems (CDSS) and evaluating its impact on early intervention, antibiotic stewardship, and long-term patient outcomes will be important steps toward the study’s implementation in routine practice. From a medical informatics perspective, the LGBM model this study propose here has good potential for real-world applications. Given that the model only includes routinely collected clinical, laboratory, and radiological variables, it could be easily integrated into hospital information systems or neurocritical CDSS for real-time intracranial infection after ICH risk assessment. Early preventive interventions may be implemented in a neurocritical care setting as a result, which may enhance resource allocation and improve patient outcomes.

## Conclusion

In conclusion, the LGBM-based model showed good predictive performance for ICI after ICH and stable results in internal validation. The predicted value was independently associated with infection risk and 180-day functional outcome, suggesting potential prognostic relevance. This model may serve as a useful tool for early risk stratification, but external validation in independent cohorts is still required before broader clinical implementation.

## Data Availability

The raw data supporting the conclusions of this article will be made available by the authors, without undue reservation.
