# Supplementary material for: Development and internal validation of a machine learning model for predicting intracranial infection after spontaneous intracerebral hemorrhage: a two-center retrospective study
Source: Front Neurol. 2026 May 4;17:1835984. doi: 10.3389/fneur.2026.1835984 (PMC13180598; doi:10.3389/fneur.2026.1835984)
Supplement: Supplementary file 1 [file Supplementary_file_1.docx]

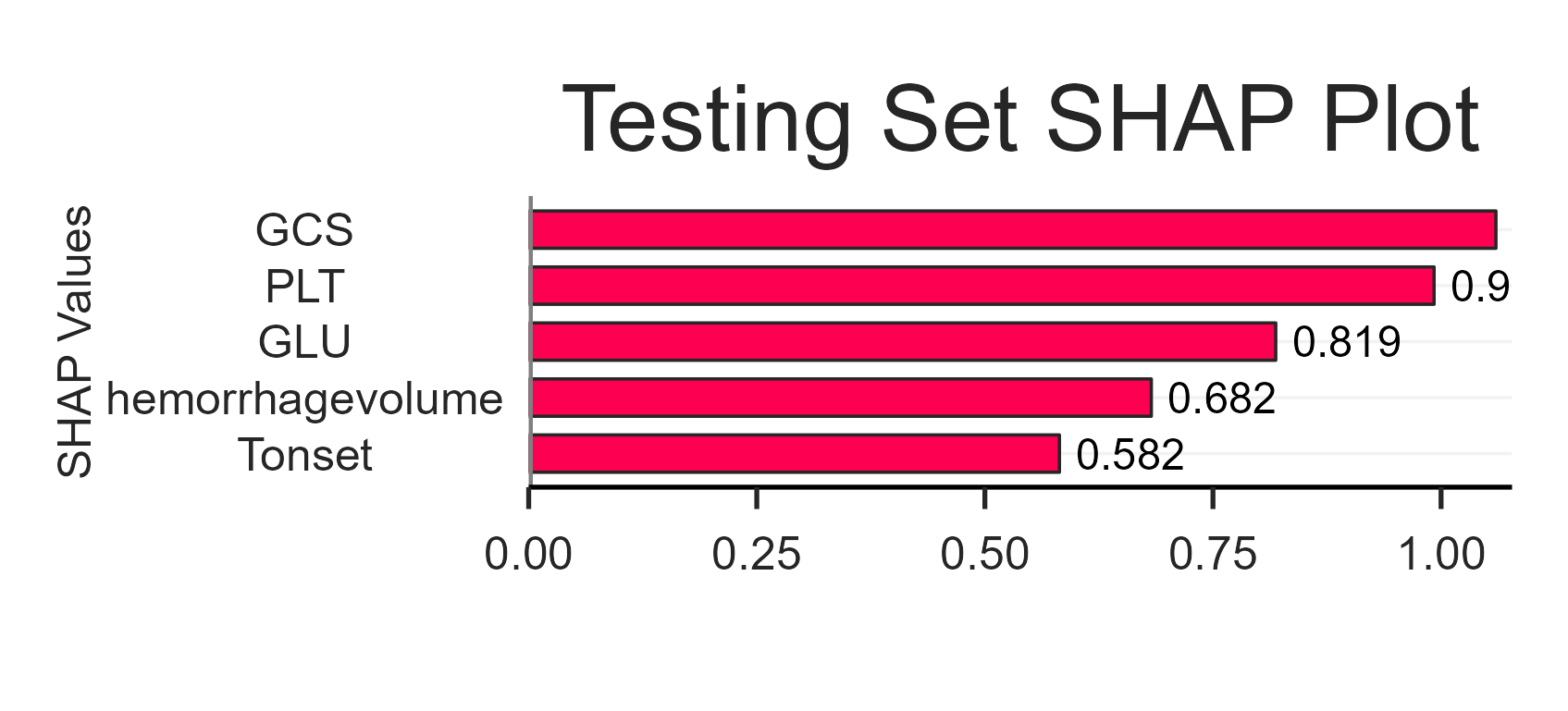


**Supplementary Figure S1 SHAP-based interpretability analysis of the LGBM model in the testing set**

The bar plot shows the global contribution of the selected variables to the LGBM model output based on mean absolute SHAP values in the testing set. GCS had the highest contribution, followed by PLT, GLU, hemorrhage volume, and time to onset, indicating that these variables were the principal drivers of model prediction in the testing set.
